# Janus Biopolymer Sponge with Porous Structure Based on Water Hyacinth Petiole for Efficient Solar Steam Generation

**DOI:** 10.3390/ijms23169185

**Published:** 2022-08-16

**Authors:** Junying Li, Sheng Chen, Cuihuan Li, Mengyao Cao, Jiahui Mu, Haq Nawaz, Zhe Ling, Feng Xu

**Affiliations:** 1 Beijing Key Laboratory of Lignocellulosic Chemistry, Beijing Forestry University, Beijing 100083, China; 2 State Key Laboratory of Pulp and Paper Engineering, South China University of Technology, Guangzhou 510640, China; 3 Jiangsu Co-Innovation Center of Efficient Processing and Utilization of Forest Resources, College of Chemical Engineering, Nanjing Forestry University, Nanjing 210037, China

**Keywords:** Janus, biopolymer sponge, porous structure, water hyacinth, solar steam generation

## Abstract

Solar-driven steam generation for desalination is a facile, sustainable, and energy-saving approach to produce clean freshwater. However, the complicated fabrication process, high cost, potential environmental impact, and salt crystallization of conventional evaporators limit their large-scale application. Herein, we present a sustainable Janus evaporator based on a biopolymer sponge from the water hyacinth petiole (WHP) for high-performance solar steam generation. The freeze-dried WHP maintained its original porous structure and aligned channels well, and therefore holds the capability for rapid water transport due to strong capillary action. The WHP coated with carbon nanotubes/ethyl cellulose paste on its surface (WHP-C) gains a good photothermal property, thus achieving an efficient solar steam generation with a rate of 1.50 kg m^−2^ h^−1^ under 1 sun irradiation. Moreover, the WHP-C after hydrophobic modification by fluorocarbon (WHP-CH) is endowed with high water repellency and exhibits good salt resistance during long-term solar desalination. Additionally, we demonstrate that a stable wet surface that enables efficient water supply and vapor escape is also significant to the successive desalination of a solar evaporator. Our work provides new insights into the high-value utilization of biomass waste, i.e., water hyacinth, and the development of sustainable interfacial solar evaporators for the environmentally friendly production of freshwater.

## 1. Introduction

In recent decades, with rapid population growth and increasingly severe water pollution, the scarcity of clean freshwater has attracted great concern as a common issue in the world. To produce freshwater from unconventional water sources including seawater, brackish groundwater, and so forth, diverse water treatment technologies such as forward osmosis [1], reverse osmosis [2], membrane distillation [3], and nanofiltration [4] have been developed. Among the methods for desalination from brine, solar-driven evaporation is a facile, sustainable, and energy-saving approach that does not require electricity infrastructure and is modularizable for easy deployment [5,6,7]. However, the efficiency of steam vapor generation for a traditional solar evaporator was limited due to the heat energy from sunlight not being sufficient, and most of it was used to directly heat the bulk water, thus causing serious heat loss [8,9]. To address this issue and improve solar steam generation, an interfacial solar-driven evaporator that can localize solar energy at the water–air interface was proposed to achieve low-cost, efficient, and sustainable production of clean freshwater from brine [9,10,11]. Note that the photothermal materials that have the capability to convert solar energy into heat for water evaporation were always on the surface of or separated from the bulk water in the interfacial evaporation system. Therefore, the heat converted from solar energy was confined to the water–air interface and directly utilized to evaporate surface water, avoiding severe heat loss to bulk water.

In addition to minimized heat loss and efficient photothermal energy conversion, sufficient water transportation to the water–air interface and effective inhibition of salt crystallization are also critical to the efficient solar steam generation for long-term desalination in practice. Many attempts have been made to meet the above expectations by constructing the unique evaporator with a Janus structure, that is, preparing two-sided materials with different properties such as wettability, i.e., an asymmetric material [12,13,14]. Although these Janus solar evaporators exhibited a good performance for desalination, the sustainability and biodegradability of evaporator materials should also raise concerns. Solar evaporators mainly made of petroleum-based materials generally have a complicated fabrication process, high cost, high carbon footprint, and high environmental impact, which limits their paratactical application on a large scale. Consequently, various common biomass materials such as wood [15], bamboo [16], corn cobs [17], lotus seedpods [18], lotus leaf [19], straw [20,21,22], loofah [23,24], grasses [25,26], sugar cane [27], mushroom [28], sunflower [29], and potato [30], have been used to construct sustainable, low-cost, environmentally friendly, and highly efficient solar evaporators via facile fabrication strategies.

Water hyacinth, *Eichhornia crassipes*, a tropical monocotyledonous aquatic species of the Pontederiaceae family (pickerelweeds) that floats on water and has thick leaves, has the ability to persist in water and exhibits dramatically rapid growth: a single water hyacinth plant can produce 2.7 new plants within a week [31]. As a result, water bodies are often clogged by the dense, impermeable mats made of large colonies of water hyacinth. This can cause plenty of environmental problems such as fish populations, irrigation, biodiversity, water quality, and water transportation, which has garnered global attention [31,32,33]. Although water hyacinth has a huge yield, varying within the range of 60–150 tons per hectare, and advantages of fast growth, abundance, low cost, renewability, and sustainability, 60–75% of water hyacinth remains as waste [33,34]. Therefore, water hyacinth is a promising alternative, instead of petroleum-based products, for advanced applications in various fields such as bioenergy, compost, and evaporators as raw materials.

Herein, we prepared a Janus biopolymer sponge based on the water hyacinth petiole (WHP) for efficient solar-driven steam generation via a facile method. The obtained WHP after freeze-drying maintained its original porous structure and aligned channels well, which had the capability for rapid water transport due to strong capillary action. By further coating the paste made of carbon nanotubes (CNTs) and ethyl cellulose (EC) on the surface of the WHP, the obtained WHP-C was endowed with a good photothermal property and thereby achieved an efficient solar steam generation with a rate of 1.50 kg m^−2^ h^−1^ under 1 sun irradiation. Furthermore, the WHP-C after hydrophobic modification (WHP-CH) that had a large water contact angle demonstrated good salt resistance during a long-term solar desalination.

## 2. Results and Discussion

### 2.1. Fabrication and Morphology of Janus Sponge

**Figure 1:**
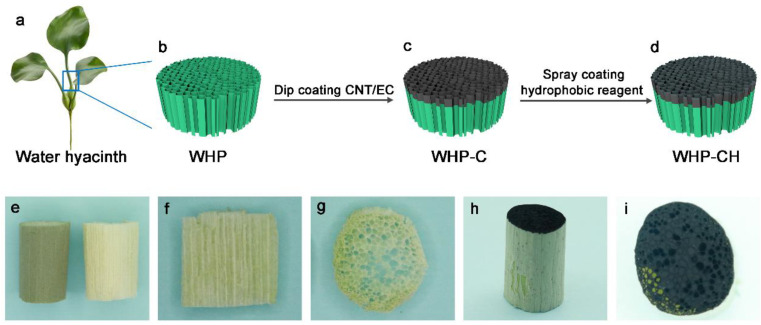
(**a**) Photo of water hyacinth petiole. (**b**–**d**) Schematic illustration showing the fabrication of WHP-CH. Photos of (**e**) WHP with (left) and without (right) epidermis, (**f**) axial-sectional WHP, (**g**) radial-sectional WHP, (**h**) front-view WHP-C, and (**i**) top-view WHP-CH.

The fabrication process of the Janus biopolymer sponge based on water hyacinth is illustrated in Figure 1a–d. As can be seen, the water hyacinth petiole (WHP) was cut to a specific shape and freeze-dried for further utilization. After dip-coating the CNT/EC paste on the top surface, the WHP-C monolith with a Janus structure was obtained and expected to be used for efficient solar-driven steam generation. Subsequently, fluorocarbon resin, a hydrophobic reagent, was introduced on the surface of the WHP-C by spray-coating to construct a more hydrophobic surface, which is beneficial to the salt resistance of the WHP-CH during solar-driven desalination.

The surface morphology of the WHPs with and without an epidermis is shown in Figure 1e. The WHP with an epidermis was green; however, after removing the epidermis, the WHP exhibited a pale-yellow color. From the axial-sectional WHP (Figure 1f), we observed the vertically aligned channels, which are critical to the efficient water transportation of water hyacinths in growth. Furthermore, the radial-sectional WHP showed an open porous structure (Figure 1g), indicating that the vertical channels were throughout the whole WHP. After dip-coating the paste of CNT/EC, the Janus WHP-C was obtained and presented a significantly dark top surface (Figure 1h), which was expected to achieve an excellent photothermal property for potential application in solar-driven steam generation. By further hydrophobic modification using fluorocarbon reagent, the as-prepared WHP-CH exhibited no obvious morphological changes on its surface and the porous structure was well-maintained (Figure 1i).

**Figure 2:**
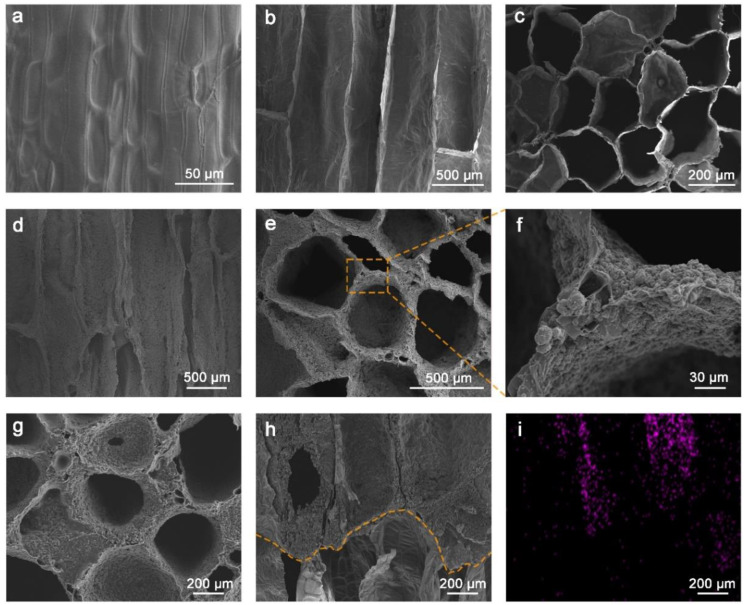
SEM images of (**a**) WHP with epidermis, (**b**) axial-sectional WHP, (**c**) radial-sectional WHP, (**d**) axial-sectional WHP-C, (**e**,**f**) radial-sectional WHP-C, (**g**) radial-sectional WHP-CH, (**h**) axial-sectional WHP-CH. (**i**) EDS mapping showing the distribution of fluorine (F) element in (**h**).

The microstructures of the biopolymer sponge were further characterized by SEM images (Figure 2). As can be seen, the epidermis of the WHP had a rough surface, and no pores were observed (Figure 2a). Inside the WHP, vertically aligned channels with a width of 200–500 μm were demonstrated in the SEM image of the axial-sectional WHP (Figure 2b). The open porous structures, namely the aerenchyma tissue of the WHP, were also observed in the SEM image of the radial-sectional WHP (Figure 2c). Note that the surface morphology of pore walls inside the WHP was relatively smooth and flat. Interestingly, some of the pore walls of the WHP were also composed of vertically aligned channels, i.e., parenchymatous cells (Figure S1), revealing the hierarchical porous structure of the WHP. This is significant for the WHP to achieve rapid water transport, which is beneficial for the application of the WHP in solar-driven vapor generation. As shown in Video S1 and Figure S2, the filter paper on the top of the WHP became wet quickly, 30 s after the bottom of the WHP was immersed in water, due to the capillary action of aligned channels in the WHP.

After dip-coating the CNT/EC paste, the as-prepared WHP-C exhibited a highly rough surface morphology, which could be seen in SEM images of both the axial-sectional WHP-C (Figure 2d) and the radial-sectional WHP-C (Figure 2e,f). In addition, the pore walls of the WHP-C were significantly thickened when compared to that of the WHP, due to the coating of CNT/EC. The WHP-CH exhibited no obvious morphological changes after hydrophobic modification via fluorocarbon reagent, as shown in the SEM image of the radial-sectional WHP-CH (Figure 2g). To further evaluate the distribution of hydrophobic reagent inside the WHP-CH after spray-coating, the SEM/EDS mapping of the axial-sectional WHP-CH was captured and shown in Figure 2h,i, and the related spectra are shown in Figure S3. The orange dash line in Figure 2h indicates the boundary between CNT/EC (top) and the WHP (bottom). As observed, the distribution of fluorine elements was consistent with the area of channels, indicating that the fluorocarbon reagent was sprayed into the pores from the top of the WHP-CH.

### 2.2. Chemical Structure and Mechanical Property

**Figure 3:**
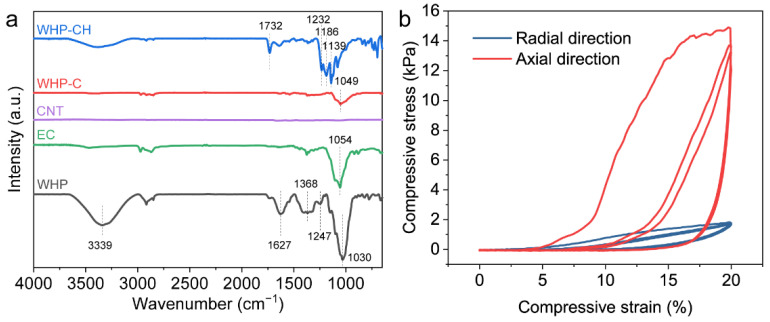
(**a**) FTIR spectra of WHP, EC, CNT, WHP-C, and WHP-CH. (**b**) Compressive stress–strain curves of WHP during loading–unloading cycles (3 times) in radial and axial direction, showing the anisotropic feature of WHP.

The chemical structure of the WHP, WHP-C, and WHP-CH were characterized by Fourier transform infrared (FTIR) spectroscopy, and the results are shown in Figure 3a. The spectrum of the WHP showed a typical O-H stretching peak of around 3339 cm^−1^ due to the identical chemical groups of lignocellulosic biomass [35,36,37]. Additionally, the main FTIR peaks of the WHP were observed at 1030 cm^−1^ (attributed to the C-O stretching) and 1627 cm^−1^ (corresponded to the C=O stretching due to hemicellulose) [36]. The peak at 1247 cm^−1^ corresponded to ester, ether, or phenol compounds, and the peak at 1368 cm^−1^ corresponded to C-H bending vibration [33]. The EC exhibited a sharp peak at 1054 cm^−1^, which could be attributed to the C-O stretching vibration [38], while no obvious peak was observed for the FTIR spectrum of CNT. After applying the coating of CNT/EC, the main FTIR peak intensity of the WHP-C decreased due to the pure carbon material, i.e., CNT. However, the peak at 1049 cm^−1^, which is attributed to the C-O stretching vibration, was observed due to EC. By further hydrophobic modification, the obtained WHP-CH exhibited adsorption peaks at 1139, 1186, and 1232 cm^−1^, which are contributed to the C-F group of fluorocarbon reagent; additionally, the emerging peak at 1732 cm^−1^ is ascribed to the carbonyl (C=O) stretching vibration of fluorocarbon reagent [39,40,41]. The introduction of C-F groups to the WHP-CH was expected to improve its hydrophobicity, thus endowing the WHP-CH with a good salt-resistance feature during solar-driven desalination.

The mechanical properties are important for the practical applications of the WHP in various fields. As shown in Video S2 and Figure S4, the WHP was able to rebound to the original shape after compression and release, revealing its good compressibility in the radial direction; however, the WHP was relatively rigid and could not rebound to its original shape after compression and release in the axial direction. This indicated that the WHP was anisotropic due to its naturally engineered structure, i.e., vertically aligned channels. To further quantitatively evaluate the mechanical properties of the WHP, the compressive stresses as a function of strains in the radial and axial direction were measured for three cycles, and the results are shown in Figure 3b. As can be seen, the maximum stress at 20% strain for the WHP in the axial direction was 13.17–14.84 kPa, which was much higher than that for the WHP in the radial direction (only 1.65–1.83 kPa). This also reveals the anisotropy of the WHP. Moreover, obvious hysteresis loops in the loading–unloading cycles were observed for the compression of the WHP in the axial direction due to mechanical loss, which, however, was not found for the compression of the WHP in the radial direction.

### 2.3. Photothermal Property and Solar Steam Generation

**Figure 4:**
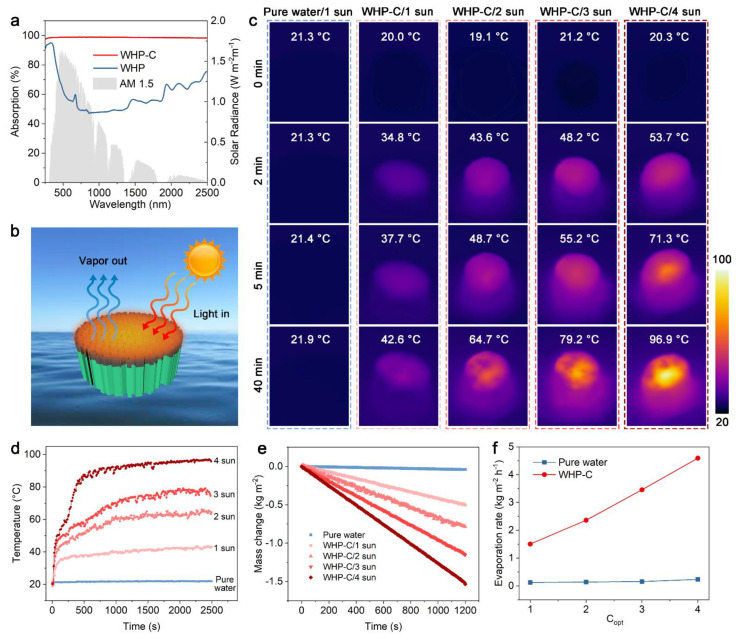
(**a**) UV-Vis-NIR spectra of WHP and WHP-C. The normalized spectral solar irradiance density of the air mass 1.5 global (AM 1.5 G) tilt solar spectrum is shown by the gray area. (**b**) Schematic illustration showing the solar-driven vapor generation by WHP-C. (**c**) Infrared images showing the temperature distribution and changes of pure water under 1 sun irradiation and the WHP-C under different intensities of light (1, 2, 3, 4 sun). The (**d**) maximum surface temperature and (**e**) mass changes of pure water under 1 sun irradiation and the WHP-C under different intensities of light (1, 2, 3, 4 sun). (**f**) The evaporation rates of pure water and the WHP-C under different intensities of light (1, 2, 3, 4 sun). C_opt_ represents the light concentration coefficient of the xenon lamp.

The solar absorption capability, namely photothermal performance, of the WHP-C is of great concern for its application as an evaporator in solar-driven steam generation. The ultraviolet–visible–near infrared (UV-Vis-NIR) spectra of the WHP and WHP-C were measured, and the results are shown in Figure 4a. The light absorption of the WHP was only 47.1–75.4% in the wavelength range of 450–2500 nm. In comparison, the WHP-C exhibited significantly efficient light absorption, averaging 98.62% over the wavelength from 200 to 2500 nm, mainly due to the addition of CNTs. Taking into consideration the excellent photothermal property of the WHP-C, it had promising potential for application in solar-driven steam generation and desalination. As illustrated in Figure 4b, the Janus porous WHP-C with aligned channels can achieve rapid water transport by capillary action and form a high-temperature region on the surface after absorbing solar illumination, thus realizing efficient steam vapor generation. The temperature change and distribution of pure water and the WHP-C floated on water under different intensities of solar irradiation (1–4 sun) were further monitored by an infrared camera, and the results are presented in Figure 4c,d. No obvious temperature change was observed for pure water under 1 sun irradiation. However, the surface temperature of the WHP-C rapidly increased from 20 to 34.8 °C within 2 min and reached a stable temperature of about 42.6 °C after 40 min under 1 sun irradiation. Moreover, the temperature of the WHP-C increased to 96.9 °C after 40 min irradiation, as the light intensity increased to 4 sun. Therefore, much heat confined on the surface of the Janus WHP-C (i.e., evaporation interface) would achieve efficient solar steam generation.

During the process of solar steam generation, the mass of the WHP-C evaporator gradually decreased over time after being exposed to different intensities of light. As shown in Figure 4e, the mass change of the WHP-C under 1 sun irradiation for 1200 s was −0.50 kg m^−2^, which increased to −1.54 kg m^−2^ as the light intensity increased to 4 sun. Nevertheless, the mass change of pure water under 1 sun irradiation for 1200 s was only −0.04 kg m^−2^. Furthermore, the solar evaporation rate of the WHP-C and pure water under different intensities of light was also measured, and the results are shown in Figure 4f. The evaporation rate of the WHP-C increased from 1.50 to 4.59 kg m^−2^ h^−1^ as the light intensity, i.e., concentration coefficient, increased from 1 to 4 sun. In comparison, the pure water exhibited a very low evaporation rate of only 0.23 kg m^−2^ h^−1^ even under 4 sun irradiation. Given the above, the Janus WHP-C with aligned channels having rapid water transportation and good photothermal property is a promising interfacial evaporator for efficient solar steam generation.

**Figure 5:**
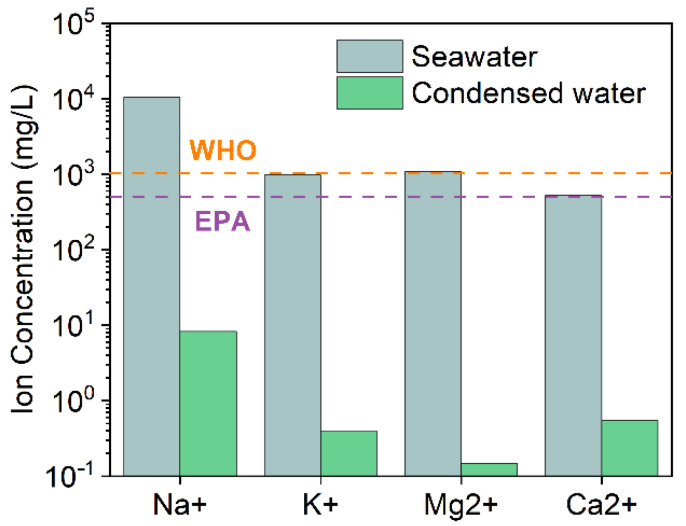
Ion concentrations of the seawater and the water condensed from solar steam generation by the WHP-C.

**Figure 6:**
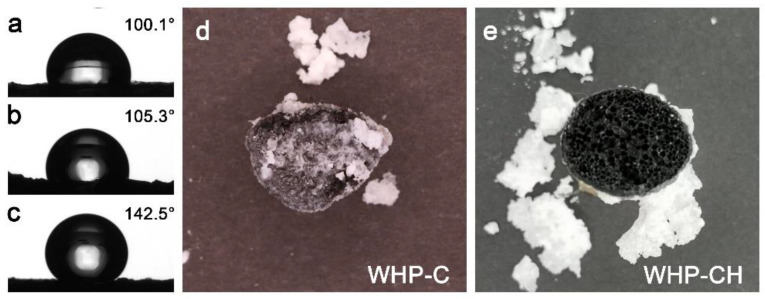
Water contact angles of (**a**) WHP, (**b**) WHP-C, and (**c**) WHP-CH. (**d**,**e**) Photos of WHP-C and WHP-CH after evaporating 30 wt% NaCl solution for 2 h under 4 sun irradiation, showing the good salt resistance of WHP-CH and the salt accumulation of the WHP-C.

The purification performance of the desalination for the WHP-C was also studied by measuring the ion concentration in condensed water from the solar steam of seawater. As shown in Figure 5, the concentration of Na^+^, K^+^, Mg^2+^, and Ca^2+^ in condensed water after a single desalination process dramatically decreased three or four orders of magnitude when compared to that in original seawater. The quality of condensed water after solar-driven desalination of seawater by the WHP-C was significantly better than the standards of drinking water according to the World Health Organization (WHO) and the U.S. Environmental Protection Agency (EPA). The surface wettability of the WHP, WHP-C, and WHP-CH were studied by water contact angles (WCAs) and the results are shown in Figure 6a–c. Compared with the WHP with a WCA of 100.1°, the WHP-C exhibited a relatively higher WCA of 105.3° due to the introduction of the EC/CNT paste. After hydrophobic modification by fluorocarbon, the WCA of the obtained WHP-CH increased to 142.5° as expected, which was beneficial to the salt resistance of the WHP-CH during solar-driven desalination. Furthermore, to evaluate the salt resistance of the evaporators before and after hydrophobic modification, the WHP-C and WHP-CH were used to evaporate 30 wt% NaCl solution for 2 h under 4 sun irradiation (Figure 6d,e). After evaporating brine, the surface of the WHP-C was covered by salt crystals due to severe salt accumulation. However, although salt crystals were deposited on the side of the WHP-CH and dropped off, the hydrophobic surface of the WHP-CH remained in its original porous structure and no obvious salt crystals were observed on this top surface. In addition, the WHP-CH was used to prepare a proof-of-concept solar desalination system to evaporate realistic seawater. As shown in Figure S5, no salt was deposited on the surface of the WHP-CH after solar evaporation of seawater for 2 days (16 h daylight). The above results indicated the good salt resistance of the WHP-CH, which was important for long-term and efficient solar steam generation in desalination.

**Figure 7:**
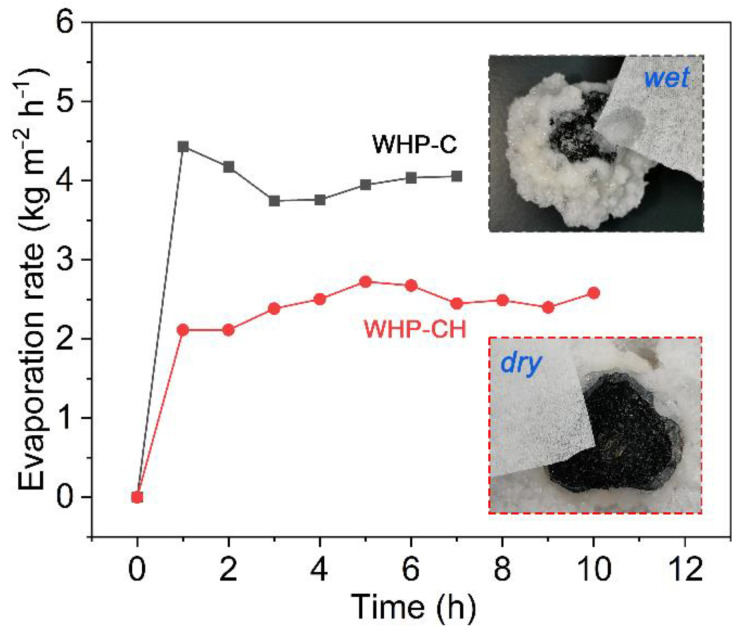
The evaporation rates of WHP-C and WHP-CH under 4 sun irradiation when treating 30 wt% NaCl solution over a long period of time. Inserts are the photos of WHP-C and WHP-CH after long-term desalination, showing a wet salt layer and a dry surface, respectively.

However, besides good salt resistance, sufficient water supply and unimpeded vapor escapes on the surface of the evaporator were also critical to stable vapor generation [42]. The evaporation rates and surface-wetting state of both WHP-C and WHP-CH during successive operation were investigated, and results are shown in Figure 7. As can be seen, the evaporation rate of the WHP-C reached 4.43 kg m^−2^ h^−1^ in 1 h and decreased by 8–14% in 3–7 h due to salt accumulation. The WHP-CH exhibited a relatively stable evaporation rate of 2.1–2.7 kg m^−2^ h^−1^ in 1–10 h. Note that, although the WHP-CH did not cause salt accumulation, it had a lower evaporation rate when compared with the WHP-C. This could be explained by their surface wetting state during the successive desalination over a long period of time. As shown by inserts in Figure 7, the salt layer deposited on the WHP-C surface was wet, which enabled efficient water supply and vapor escape; however, the WHP-CH surface was very dry, which caused limited water supply and vapor escape. Therefore, there is a trade-off between increasing surface hydrophobicity to avoid salt accumulation and improving surface wetting to achieve efficient water supply and vapor escape for the successive desalination by a solar evaporator. Future work can focus on investigating the correlation between surface wettability and vapor generation of interfacial solar evaporators.

## 3. Materials and Methods

### 3.1. Materials

Water hyacinth was collected from Kunming, China. Carbon nanotubes (CNTs) were purchased from Xianfeng Nanotechnology, Nanjing, China. Ethyl cellulose (EC, 270–330 mPa·s), ethanol (99.7%), and sodium chloride (NaCl, 99%) were purchased from Macklin, Shanghai, China. Fluorocarbon resin was provided by Sino-Fluorine Science and Technology, Shenzhen, China. All materials and reagents were used without further purification.

### 3.2. Preparation of Janus Solar Evaporator

The water hyacinth petiole (WHP) was cut and freeze-dried for 48 h in a freeze dryer (Alpha 1–2 LDplus, Christ, Osterode, Germany) to obtain the porous WHP sponge. To prepare the photothermal layer, EC and CNTs (mass ratio = 1:1) were dissolved/dispersed in ethanol with a solid content of 12 wt% by vigorous stirring. By further coating this composite CNT/EC paste on the radial-sectional surface of the WHP, the WHP coated with CNT (WHP-C), namely a Janus solar evaporator, was obtained after air-drying. The WHP-C was further spray-coated with fluorocarbon resin to obtain the WHP-C with a hydrophobic surface (WHP-CH).

### 3.3. Characterization

The microstructures of the WHP, WHP-C, and WHP-CH were characterized by a field emission SEM (Hitachi SU8010, Ibaraki, Japan), and energy-dispersive X-ray spectroscopy (EDS) elemental maps were used to demonstrate the distribution of the fluorine (F) element. The Fourier transform infrared (FTIR) spectra of samples were measured and recorded by a spectrometer (Bruker Tensor II, Billerica, MA, USA). The mechanical properties were characterized by a universal material testing machine (Zwick Roell, Ulm, Germany). The surface temperature changes were measured by an infrared thermal camera (Fotric 325 Pro, Shanghai, China). Ultraviolet–visible–near infrared spectra of samples were measured by a spectrophotometer (PerkinElmer Lambda 750, Akron, OH, USA). The thermogravimetric analyses (TGA) of samples were performed by a DTG-60 (Shimadzu, Kyoto, Japan).

### 3.4. Solar Evaporation Generation Experiment

A xenon lamp (CEL-HXUV300, CeAulight, Beijing, China) with an AM 1.5 G filter was used as the solar simulator to conduct the solar-driven steam generation experiment; the environment temperature and relative humidity were 20 °C and 40%, respectively. The photo flux of the incident light was measured by a spectroradiometer (CEL-NP2000, CeAulight, China). The WHP-C or WHP-C was placed on the pure water or saltwater surface without any support in a PTFE beaker. The mass change in water in the beaker was monitored by an electronic balance (BCE224-1CCN, Sartorius, Göttingen, Germany), which was connected to a computer using homemade software.

## 4. Conclusions

In summary, we reported a sustainable Janus evaporator based on the WHP and EC/CNT paste for efficient solar steam generation. As a biopolymer sponge, the WHP after freeze-drying maintained its porous structure and aligned channels well, which endowed the WHP with strong capillary action and thus rapid water transport. After coating EC/CNT on the surface, the obtained WHP-C had a good photothermal property and achieved high-performance solar steam generation with an evaporation rate of 1.50 kg m^−2^ h^−1^, indicating that the WHP is a promising prospect as a cost-effective raw material for a sustainable interfacial solar evaporator. Furthermore, for long-term solar desalination, the WHP-CH after hydrophobic modification gained high water repellency and exhibited good salt resistance; however, the WHP-C deposited with a wet salt layer had a higher evaporation rate due to sufficient water supply and vapor escape. These findings offered new insights into the utilization of water hyacinth waste to reduce related environmental problems in water bodies and the development of sustainable interfacial solar evaporators for freshwater production.

## Data Availability

The data presented in this study are available on request from the corresponding authors.

## Supplementary Materials

The following supporting information can be downloaded at: https://www.mdpi.com/article/10.3390/ijms23169185/s1.
