# Supplementary material for: Janus Biopolymer Sponge with Porous Structure Based on Water Hyacinth Petiole for Efficient Solar Steam Generation
Source: Int J Mol Sci. 2022 Aug 16;23(16):9185. doi: 10.3390/ijms23169185 (PMC9408865; doi:10.3390/ijms23169185)
Supplement: Supplementary file 1 [file ijms-23-09185-s001.zip › Revised Supporting Information.pdf]

Supporting Information

Janus biopolymer sponge with porous structure based on water hyacinth petiole for efficient solar steam generation

Junying Li [1], Sheng Chen [1,2,*], Cuihuan Li [1], Mengyao Cao [1], Jiahui Mu [1], Haq Nawaz [1], Zhe Ling [3] and Feng Xu [1,*]

1 Beijing Key Laboratory of Lignocellulosic Chemistry, Beijing Forestry University, Beijing 100083, China

2 State Key Laboratory of Pulp and Paper Engineering, South China University of Technology, Guangzhou 510640, China

3 Jiangsu Co-Innovation Center of Efficient Processing and Utilization of Forest Resources, College of Chemical Engineering, Nanjing Forestry University, Nanjing 210037, China

* Correspondence: shengchen@bjfu.edu.cn (S.C); xfx315@bjfu.edu.cn (F.X.)

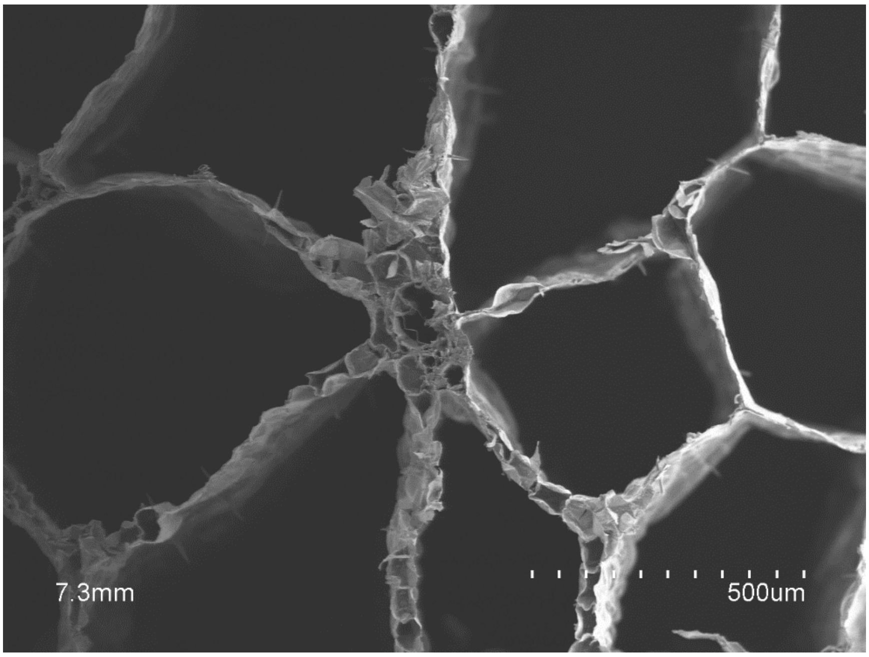

Figure S1. SEM image of the radial-sectional WHP showing the hieratical porous structures.

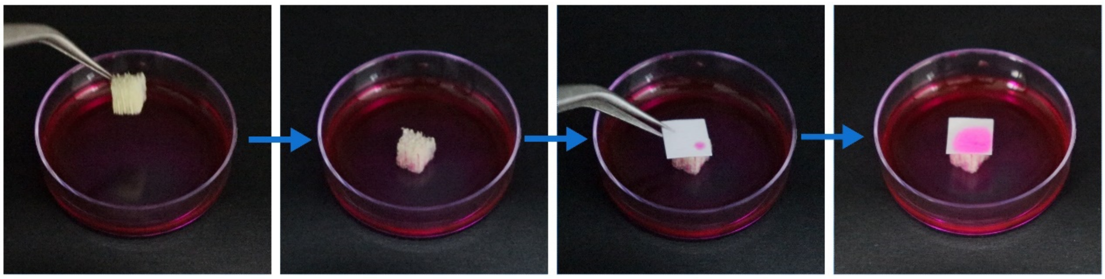

Figure S2. Photos showing the rapid water transportation through the vertically aligned channels of WHP. Filter paper was put on the top surface of WHP to visualize the surface water.

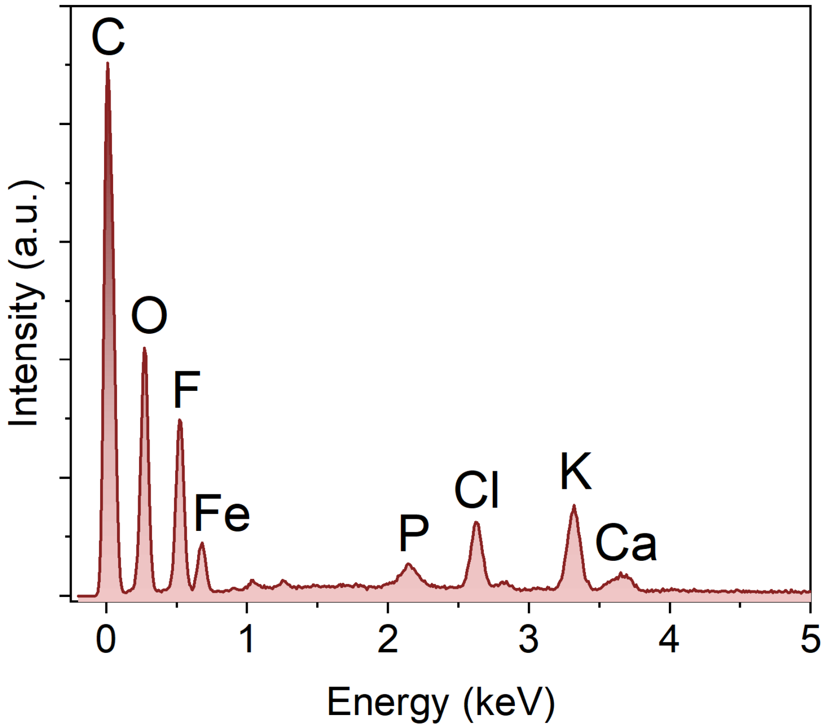

Figure S3. The EDS spectra of WHP-CH.

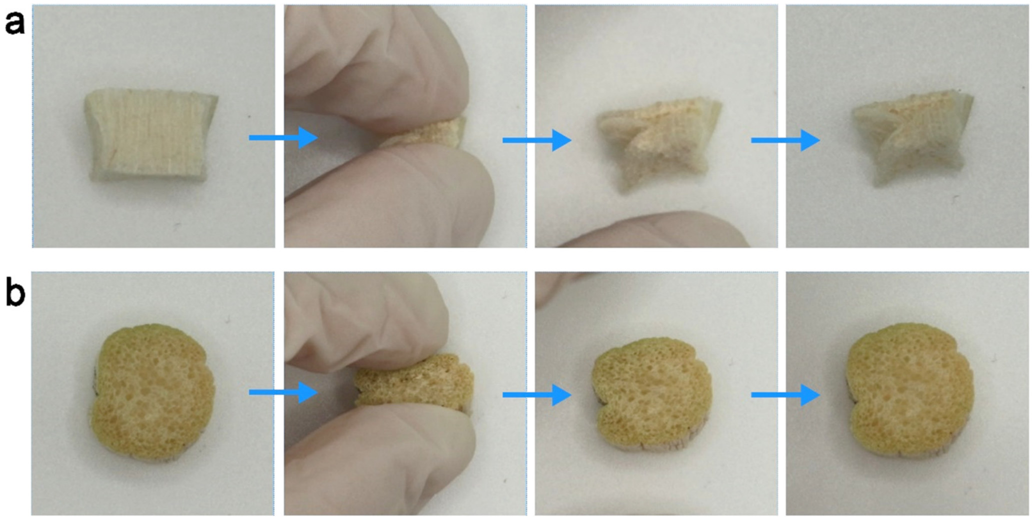

Figure S4. Photos of the WHPs under compression and release in (a) axial and (b) radial direction, showing the anisotropy of the WHP.

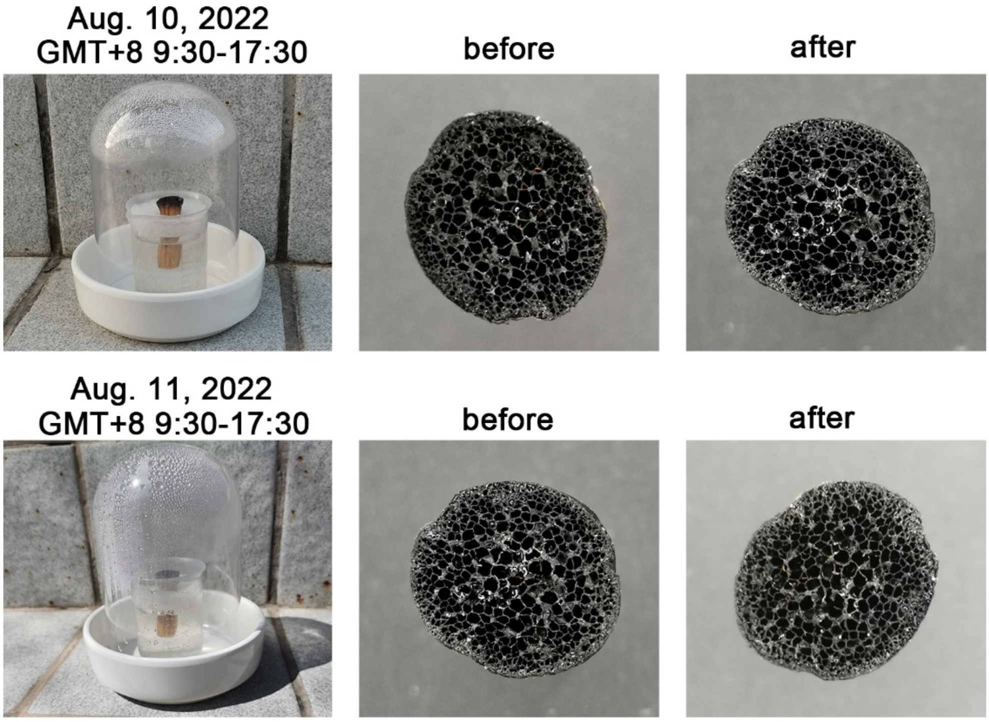

Figure S5. Photos of the WHP-CH solar desalination system and the surface of WHP-CH before and after long-term evaporating realistic seawater for 2 days (16-h daylight).
